# Tick-Borne Encephalitis Virus in Ticks and Roe Deer, the Netherlands

**DOI:** 10.3201/eid2306.161247

**Published:** 2017-06

**Authors:** Setareh Jahfari, Ankje de Vries, Jolianne M. Rijks, Steven Van Gucht, Harry Vennema, Hein Sprong, Barry Rockx

**Affiliations:** National Institute for Public Health and the Environment, Bilthoven, the Netherlands (S. Jahfari, A. de Vries, H. Vennema, H. Sprong, B. Rockx);; Utrecht University, Utrecht, the Netherlands (J.M. Rijks);; Scientific Institute of Public Health, Brussels, Belgium (S. Van Gucht)

**Keywords:** tick-borne encephalitis, meningitis/encephalitis, ticks, roe deer, the Netherlands, tick-borne encephalitis virus, viruses, Capreolus capreolus, Ixodes ricinus, TBEV, TBEV-EU, flavivirus, zoonoses, vector-borne infections

## Abstract

We report the presence of tick-borne encephalitis virus (TBEV) in the Netherlands. Serologic screening of roe deer found TBEV-neutralizing antibodies with a seroprevalence of 2%, and TBEV RNA was detected in 2 ticks from the same location. Enhanced surveillance and awareness among medical professionals has led to the identification of autochthonous cases.

Tick-borne encephalitis virus (TBEV) can infect humans, causing febrile illness; neurologic complications include encephalitis (*1*). TBEV is transmitted through bites of infected ticks to many animals, including deer, which serve as feeding hosts for ticks (*2*,*3*). Expansion of TBEV subtypes has been reported (*4*). Reports of TBEV-neutralizing antibodies in wildlife and cattle in Belgium prompted us to reinvestigate the presence of TBEV in the Netherlands (*5*,*6*).

During January–September 2010, hunters collected 297 blood samples from roe deer (*Capreolus capreolus*) from locations across the Netherlands. We used a commercial ELISA to detect TBEV-reactive antibodies in roe deer serum samples. Serologic screening of all 297 samples by ELISA yielded 6 positive and 8 borderline results. All positive, 7 borderline, and 3 negative serum samples were confirmed by testing in a TBEV serum neutralization test (SNT), with the Neudörfl strain as the accepted prototype TBEV-EU, formerly called central European encephalitis virus (*5*). Five of 6 ELISA positive samples and 1 of 7 borderline samples were confirmed positive by SNT. Five of the 6 SNT-confirmed roe deer were shot at or near a popular recreation area, the National Park Sallandse Heuvelrug (Figure, panel A).

**Figure:**
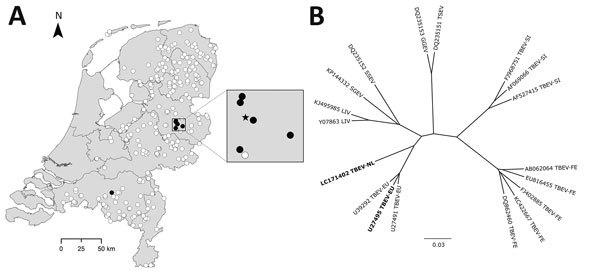
Spatial distribution of TBEV-positive roe deer and genetic cluster analysis of TBEV sequences from the Netherlands. A) Spatial distribution of serologic test results (solid black circle, SNT positive; open white circle, ELISA and/or SNT negative) for 297 serum samples from roe deer collected according to a sampling scheme designed to obtain a representative sample of the roe deer population from locations across the Netherlands. Enlargement of the National Park Sallandse Heuvelrug area indicates the locations of the TBEV serologically positive roe deer (solid black circle) in relation to the site with reverse transcription quantitative PCR–positive ticks (solid black star) from 2015. B) Genetic cluster analysis of TBEV-NL sequences obtained from tick pools in the Netherlands with other tickborne viruses (indicated by GenBank accession number). Bold indicates the TBEV-NL sequence, which consists of 10,242 nt of the genome (GenBank accession no. LC171402), and the TBEV-EU strain with which TBEV-NL clusters. Where available, representatives of the subtypes are included. We conducted distance-based analyses using Kimura 2-parameters distance estimates and constructed the trees using the neighbor-joining algorithm, implemented in Bionumerics 7.1 (Applied Math, Sint-Martens-Latem, Belgium). We calculated bootstrap proportions by analyzing 1,000 replicates for neighbor-joining trees. Scale bar indicates nucleotide substitutions per site. GGEV, Greek goat encephalomyelitis virus; LIV, Louping ill virus; SGEV, Spanish goat encephalitis virus; SSEV, Spanish sheep encephalitis virus; TBEV, tickborne encephalitis virus; TBEV-EU, TBEV European subtype; TBEV-FE, Far Eastern subtype; TBEV-NL, TBEV Netherlands subtype; TBEV-SI, TBEV Siberian subtype; TSEV, Turkish sheep encephalomyelitis virus.

In response to the serologic findings, we collected 1,160 nymph and 300 adult *Ixodes ricinus* ticks by blanket dragging in 7 locations at the national park in September 2015. We extracted RNA from pools of 5 nymphs or 2 adults (*7*) and tested for flavivirus by using a reverse transcription quantitative PCR. We detected flavivirus RNA in 1 nymph pool and 1 pool of adult female ticks.

To obtain sequences of the 2 reverse transcription quantitative PCR–positive samples, we used primers and protocols as described (*8*). Both sequences obtained from the tick pools were identical. The sequences obtained in this study were designated TBEV-NL and clustered within the TBEV-EU subtype complex (Figure, panel B), with a 91% sequence identity with the currently known TBEV-EU sequences.

 TBEV-EU RNA in 2 pools of ticks collected through surveillance in 1 national park confirms the presence of TBEV-EU in the Netherlands. Serologic evidence that roe deer from the same location had been infected with a flavivirus, most probably a TBEV, 5 years before the detection of TBEV RNA in ticks suggests that TBEV has been endemic to the Netherlands for at least 5 years.

The concentration of serologically positive roe deer is striking and remains unexplained. One explanation could be that this area has dense beech tree coverage, and beechnuts are a major food source for roe deer and the bank vole (*Myodes glareolus*)*.* These host species play a pivotal role in the TBEV enzootic cycle; a habitat suitable for both may have enhanced the local establishment and spread of TBEV. In addition, the finding of a serologically positive roe deer in a southern province of the Netherlands (Figure, panel A), also known for the presence of beech trees, suggests that TBEV is distributed more widely within the Netherlands.

Dissemination of information about the occurrence of TBEV in ticks and wildlife is needed for medical professionals and the general public. In response to our findings, 2 autochthonous TBEV infections were reported in the Netherlands (*9**,**10*). At least 1 of these autochthonous cases was infected with a TBEV strain showing 99% homology with the Neudörfl strain, suggesting the presence of multiple TBEV-EU strains in the Netherlands. Our findings indicate that clinicians should be aware of the possibility for TBEV infection in humans in the Netherlands.
